# Tuberculosis—The Next Plague?

**Published:** 1992-08

**Authors:** Ben J. L. Burton

**Affiliations:** Clinical Medical Student U.C.M.S.M., London


### Tuberculosis — the next plague?

**Ben J. L. Burton**
Clinical Medical Student
U.C.M.S.M., London

Many doctors in the U.K. are quietly complacent about tuberculosis, regarding it as a disease which will shortly be eradicated. Yet recent trends in USA and Africa suggest that tuberculosis could soon return as a greater scourge than ever before.[1,2]

The dramatic resurgence in the prevalence of tuberculosis in Africa has been described as "one of the greatest public-health disasters since the bubonic plague",[3] and at a recent W.H.O. Conference on tuberculosis, Africa was described as 'lost'.[3] This implied that the tuberculosis epidemic which is currently sweeping through Africa is so immense that the situation is beyond control and current resources are totally inadequate to deal with the problem.

The cause of this African disaster is HIV-l infection, which greatly reduces resistance to infection with Mycobacteria, so that tuberculosis is now emerging as a major killer in African AIDS patients. The risk of tuberculosis is greatly increased both in asymptomatic HIV positive subjects and in those with full-blown AIDS, and 30 to 60 % of tuberculosis patients in sub-Saharan Africa are co-infected with HIV.[4] There are currently at least 2.4 million tuberculosis cases co-infected with HIV in Africa,[3] and the prevalence of TB is greatest in those areas which are worst hit by the HIV pandemic, namely East Africa, Zaire; Zambia and Burundi. It is estimated that there will be 250,000 new cases of tuberculosis in Uganda alone in the next 5 years.[4]

The problem is compounded by the fact that in HIV infected patients, spontaneous recovery from Mycobacterial infection rarely if ever occurs. Moreover there are diagnostic difficulties, since many HIV patients with pulmonary tuberculosis do not produce sputum and those who do often have negative smears, so that sputum examination is unreliable. Tuberculin skin tests are positive in only about one third af patients with tuberculosis and AIDS[5] and even the chest radiograph may show little change, or there may be atypical involvement of the middle and lower zones.[6] Given the magnitude of the problem, there is no wonder that the African authorities cannot provide the necessary contact tracing and anti-tuberculosis drugs. Neither money nor staff are available to provide even a 2 month course of anti-tuberculosis chemotherapy for this huge number of patients, and Stanford had argued that the best hope may be to try a 7 day course of drugs followed by immunotherapy, using killed Mycobacterium vaccae.[3,7]

Will there be a similar explosion of tuberculosis cases in the western world as the incidence of HIV infection rises? Obviously there are many other factors in Africa such as poor nutrition and over-crowding which predispose to tuberculosis, and it has recently been shown that even in identical social conditions, blacks are more susceptible to infection with tuberculosis than white people. A long-term follow-up study [8] of residents in racially integrated nursing homes in Arkansas showed that when both black and white people are exposed to tuberculosis, the blacks have about twice the relative risk of whites of becoming infected, though once infected, both races are equally likely to progress to clinical disease.

Accepting that there are social and racial factors which predispose to a greater risk of tuberculosis in Africa, the recent figures from USA still suggest that similar problems could easily occur in our impoverished inner cities, and great vigilance will be required if the tuberculosis problem is to be contained.

Until very recently, mortality from tuberculosis in Europe and USA had been declining for many years. This decline predated the introduction of effective chemotherapy in the 1950s, which merely accelerated it.[9] This welcome trend continued until about 1986, when the first increase in mortality rate of 3% occurred in USA.[10] The rise continued, and has been most marked in states and cities with the greatest numbers of AIDS patients. In New York City, reported cases of tuberculosis rose by 38% (from 2454 cases to 3520) in 12 months from 1989 to 1990.[11] A review of 48,000 AIDS patients between 1987 and 1989 found that 2.5% of them had extra-pulmonary tuberculosis,[12] and the incidence of tuberculosis in HIV positive individuals is 500 times that in the general population.[13]

Although AIDS has clearly played a part in this resurgence of tuberculosis in USA, other factors may be involved. Only about 10% of tuberculosis patients in USA have HIV infection, and the incidence of tuberculosis has also increased rapidly in foreign-born Asians and Pacific islanders[14] who are not in high-risk groups for HIV infection, although as recent immigrants they are, of course, likely to suffer from poor housing, overcrowding and all the other hazards of inner-city deprivation. Many other factors increase the risk of tuberculosis. These include silicosis,[15] malignancy and cytotoxic therapy,[16] end-stage renal failure and haemodialysis,[17] post-gastrectomy and jejuno-ileal bypass operations,[18] diabetes mellitus, very low body weight (compared to the ideal)) and smoking.

Taking all these factors into account however, HIV still seems the most likely cause of the recent increase in tuberculosis in USA. A recent study showed that 28% of new adult cases of tuberculosis in San Francisco were HIV positive [19] and some physicians in USA now routinely counsel their new tuberculosis patients to have a test for HIV. Prophylactic anti-tuberculosis chemotherapy is also considered for HIV positive patients who are thought to be at high risk of exposure to tuberculosis contacts.

As yet, there is little evidence of this problem in U.K., though the incidence of tuberculosis increased by 1.5% in 1988 and by a further 5.3% in 1989, but this rise was seen mainly in patients who originated from the Indian sub-continent.[20] Only 1% of British AIDS patients are of Asian origin, and the lowest rate of tuberculosis occurs in young adult white males, who have the highest rate of AIDS. Our AIDS patients tend to develop infection with Mycobacterium avium intracellulare complex (MAIC) rather than M. tuberculosis, and this characteristically develops in late-stage AIDS, not in asymptomatic HIV positive subjects.[21]

Although no clear association between HIV and M. tuberculosis has yet been established in U.K.,[21] tuberculosis occurred in 6% of 207 patients with AIDS seen at St. Mary's Hospital in London between 1983 and 1988.[22] Most authorities agree that HIV infection is likely to increase in U.K., and to spread further into the heterosexual population, and it would be foolish to dismiss the possibility of a tubercular plague in this country. Chest physicians are here to stay.

### REFERENCES

1. Styblo K. (1991). The impact of HIV infection on the global epidemiology of tuberculosis. *Bull. Int. Union Tuberculosis Lung Dis.* **66**, 27-32.
2. Styblo K, (1989). Overview and epidemiological assessment of the current global tuberculosis situation with an emphasis on control in developing countries. *Rev. Infect. Diseases.* **11**, Supplement 2, S 339-46.
3. Stanford J. L. *et al.* (1991). Is Africa lost? *Lancet*, **338**, 557-8.
4. Harries A. D. (1990). Tuberculosis and HIV infection in developing countries. *Lancet*, **335**, 387-90.
5. Reider A. *et al.* Tuberculosis and AIDS. *Florida Archives of Internal Medicine*, **149**, 1268-73.

6. Elliott A. M., Luo N., Tembo G., Halwiindi B. *et al.* (1990). Impact of HIV infection on tuberculosis in Zambia — a cross-sectional study. *British Medical Journal*, **301**, 412-415.
7. Stanford J. L., Bahr G. M., Rook G. A. W. *et al.* (1990). Immunotherapy with Mycobacterium vaccae as an adjunct to chemotherapy in the treatment of pulmonary tuberculosis. *Tubercle*, **71**, 87-93.
8. Stead W. W., Senner J. W., Reddick W. J., Lofgren J. P. (1990). Racial differences in susceptibility to infection by Mycobacterium tuberculosis. *New Eng. J. of Medicine*, **322**, 422-7.
9. Doege T. C. (1965). Tuberculosis mortality in the USA, 1900-1960. *J.A.M.A.*, **192**, 103-6.
10. Update: (1990). Tuberculosis elimination — *United States NMWR*, **39**, 153-6.
11. Charatan F. (1991). Tuberculosis soars in New York. *Brit. Med. J.*, **303**, 209.
12. Braun M. M., Byers R. H., Heywood W. L. *et al.* (1990). AIDS and extra-pulmonary tuberculosis in the U.S.A. *Arch. Int. Med.*, **150**, 1913-16.
13. Pitchenick A. E. *et al.* (1988). Mycobacterial disease: epidemiology, diagnosis, treatment and prevention. *Clinics in Chest Medicine*, **9**, 425-41.
14. Centers for Disease Control. (1987). Tuberculosis among Asian, Pacific Islanders-USA. 1985. *M.M.W.R.*, **36**, 331-4.
15. Westerholm P. *et al.* (1986). Silicosis and risk of lung cancer or lung tuberculosis, a cohort study. *Environmental Research*, **41**, 339-50.
16. Kaplan M. H. *et al.* (1974). Tuberculosis complicating neoplastic diseases: a review of 201 cases. *Cancer* , **33**, 850-858.
17. Garcia-Leoni M. E., Martin-Scapa C.,, Rodeno P., Valderrabano F. *et al.* (1990). High incidence of tuberculosis in renal patients. *Europ. J. Clin. Microbiol. Infect. Dis.*, **9**, 283-5.
18. Snider D. E. *et al.* (1982). Jejuno-ileal bypass for obesity: a risk factor for tuberculosis. *Chest*, **81**, 531-2.
19. Theuer C. P., Hopewell P. C., Elias D. *et al.* (1990). HIV infection in tuberculosis patients. *J. Infect. Dis.*, **162**, 8-12.
20. Watson J. M. *et al.* (1991). Notifications of tuberculosis in England and Wales, 1982-1989. *Communicable Diseases Report*, **1**, R13-16.
21. Shaunak S. (1991). Tuberculosis and HIV infection. *British Journal of Hospital Medicine*, **45**, 187.
22. Helbert M., Robinson D., Buchanan D. *et al.* (1990). Mycobacterial infection in patients infected with HIV Thorax, **45**, 45-8.

